# Recent Advances in Aptamer-Based Point-of-Care Testing

**DOI:** 10.3390/s25123587

**Published:** 2025-06-06

**Authors:** Senlin Luo, Xiaotian Zhang, Yuzhuo Zhang, Xiaofeng Qu, Qiru Sun, Tianhuan Peng, Quan Yuan

**Affiliations:** 1 Department of Pediatrics, The Second Xiangya Hospital, Central South University, Changsha 410011, China; luosenlin@csu.edu.cn; 2 Molecular Science and Biomedicine Laboratory (MBL), State Key Laboratory of Chemo and Biosensing, College of Biology, College of Chemistry and Chemical Engineering, Aptamer Engineering Center of Hunan Province, Hunan University, Changsha 410082, China; xiaotian@hnu.edu.cn (X.Z.); yuzhuo@hnu.edu.cn (Y.Z.); xiaofeng@hnu.edu.cn (X.Q.); sunqiru@hnu.edu.cn (Q.S.); 3 Greater Bay Area Institute for Innovation, Hunan University, Guangzhou 511300, China

**Keywords:** aptamers, point-of-care diagnosis, colorimetric sensors, fluorescent assay, electrochemical biosensing

## Abstract

Aptamers refer to a class of oligonucleotide probes that have demonstrated remarkable capabilities beyond mere genetic coding, including the unique ability to recognize and selectively bind to specific molecular targets. Numerous advantages, including accessibility for targeting a diverse array of molecules and compatibility with different signal amplification and transduction elements, underscore the application of aptamers for delivering rapid and accurate diagnostic tests at the point of care. This review provides a comprehensive summary of the recent advances in aptamer-based point-of-care testing, especially highlighting the innovative applications of aptamers in colorimetric sensors, lateral flow assays, fluorescent biosensors, and electrochemical biosensors. Additionally, current challenges in this burgeoning field and forward-looking perspectives for aptamer-based point-of-care testing are discussed.

## 1. Introduction

The Essential In Vitro Diagnostics list (EDL) from WHO emphasizes that IVDs are essential for advancing universal health coverage, addressing health emergencies and promoting healthier populations, which are the three strategic priorities of WHO’s thirteenth general program of work covering 2019–2023 [1]. However, this goal remains far from being achieved, particularly in less developed countries with limited medical resources. Considering the limited medical resources available in centralized hospitals and laboratories which cannot meet the rapidly growing demand for healthcare services, there is widespread recognition that the development of affordable healthcare services delivered close to patients offers a preferred solution to alleviate the burdens on centralized healthcare facilities [2,3]. On this basis, the innovative point-of-care testing (POCT), with advantages including reduced turnaround time, less dependence on sophisticated equipment, ease of use with no need for professionals, and low cost, has been developed rapidly in the past decades and plays an increasingly significant role in healthcare systems [4,5,6,7]. Two of the most typical examples are the ability to analyze blood sugar levels at home using a portable glucometer and the capability to determine pregnancy within minutes using just a few drops of urine.

According to the international standard (ISO 15189:2022) [4], POCT is defined as “testing that is performed near or at the site of a patient with the result leading to possible change in the care of the patient” [4]. Similarly to typical biosensors, POCT products typically consist of three basic units: the target-recognizing ligands, the signal transducer or amplifier, and the signal detector. It is universally acknowledged that the development of specific ligands with high affinity for target molecules is an essential prerequisite to guarantee the sensitivity and specificity of POCT sensors. Influenced by traditional in vitro diagnosis (IVD) techniques, antibodies are typically used as molecular recognition elements in the commercialized POCT products. Unfortunately, the limited stability, uncontrollable batch-to-batch variation during manufacturing, and their difficulty in modification significantly hinder the performance of POCT products [8]. Furthermore, the rise of precision medicine has placed higher demands on POCT in terms of accuracy, sensitivity, and stability, thereby motivating researchers to innovate novel molecular tools for the next generation of POCT [9,10].

**Figure 1:**
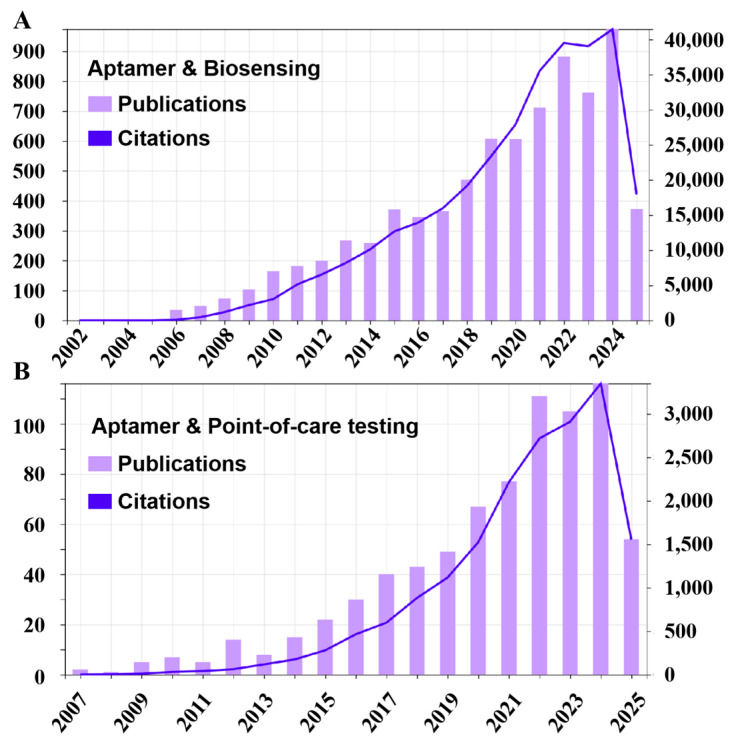
The statistical analysis of publications and citations from 2006 to 2025. (**A**) Statistic data using “aptamer & biosensing” as keywords. (**B**) Statistic data using “aptamer & point-of-care testing” as keywords. All data were sourced from the Web of Science.

Nucleic acids are inherently large biomolecules present in all living organisms, serving as the repository for genomic information. They have been extensively employed as biomarkers for molecular diagnosis. More excitingly, the discovery of aptamers has profoundly broadened the application of nucleic acid molecules for biomedical application [11,12,13,14,15]. Due to the excellent properties such as high chemical stability, being able to be chemically synthesized and modified with remarkable batch-to-batch consistency, and seamless compatibility with diverse signal amplification and transduction components, aptamers have been extensively utilized in POCT biosensors for target recognition, signal conversion, and amplification (Figure 1) [16,17,18]. In this review article, we survey major advances in aptamers-based biosensing, focusing especially on the application of aptamers in innovative point-of-care devices. We also discuss the challenges in this burgeoning field and provide a forward-looking perspective for aptamer-based point-of-care testing.

## 2. Aptamers and Systematic Evolution of Ligands by Exponential Enrichment Technology

Aptamers, also known as “chemical antibodies”, are single-stranded oligonucleotides with the capability to recognize and bind to their target molecules with high affinity and specificity [19,20,21]. As a novel type of ligand molecule, aptamers possess a series of superior properties for biomedical application [22,23,24]. Firstly, aptamers exhibit a broader spectrum of target molecules, particularly for low immunogenic targets and toxic molecules that are unable to elicit antibodies through immunoreactions. Secondly, aptamers can be easily synthesized on a large scale through solid-phase chemical synthesis technology, ensuring batch-to-batch consistency. Moreover, the development of nucleic acid chemistry enables precise modification of aptamers with a series of functional groups at specific sites. Thirdly, the formation of specific secondary and tertiary structures through intramolecular interactions serves as the structural basis for aptamers to achieve specific molecular recognition. Reversible structural changes in aptamers grant them higher thermal and chemical stability, although natural aptamers are susceptible to enzymatic degradation, thereby affecting their functions. A series of chemical modifications to enhance their resistance to nucleases have been developed and proven effective in numerous practices. Due to their exceptional properties, aptamers have found widespread application in biomedicine over the past three decades [25]. Currently, more than 50 companies worldwide are actively involved in aptamer-based clinical applications, primarily focusing on diagnostics and therapeutics. A continuously growing aptamer industry is predicted to emerge and thrive [26,27].

The aptamers were generated through an in vitro evolution process known as systematic evolution of ligands by exponential enrichment (SELEX) [28,29]. Firstly, oligonucleotide libraries with a random region flanked by two constant primer binding regions was constructed. Then, repeated positive and negative screenings were conducted through incubating the library with targets and control analogues, respectively. The enriched sequences from each screening round were amplified through polymerase chain reaction (PCR). Once the evolution reached a plateau phase, the enriched oligonucleotides were analyzed by high-throughput sequencing and bioinformatics. The candidate sequences were synthesized and evaluated to assess their affinity and specificity. Notably, other novel technologies, such as capillary electrophoresis, micro-fluidic chips, and particle display technology, have now been merged into the SELEX process, resulting in improved efficiency and simplified procedures [30,31,32,33,34,35]. Over the past three decades, aptamers targeting a diverse array of molecules have been identified and utilized to develop aptasensors based on various operational principles [36,37].

## 3. Aptamer-Based Optical Biosensors

### 3.1. Colorimetric Aptasensors

Colorimetric assays stand as one of the most popular formats for point-of-care testing, offering both qualitative and quantitative insights into various disease markers [38]. By measuring the color changes induced by a specific target analyte, these assays provide a straightforward, economical, and rapid methodology for medical diagnostics. Due to the simplicity and minimal requirement for instrumentation, colorimetric assays offer a practical and efficient solution for clinical diagnostics, especially in scenarios demanding rapid analysis and in regions with limited resources. As a superior alternative to antibodies, aptamers have been extensively employed as recognition elements in numerous traditional colorimetric assays, such as aptamer-linked immunosorbent assays or enzyme-linked aptamer assays, demonstrating comparable or even superior performance to antibody-based assays. Moreover, according to the intrinsic optical properties of gold nanoparticles (Au NPs) and specific interaction between Au NPs and single-strand aptamers, the Au NPs-based colorimetric assays have been widely applied for rapid diagnosis. In this part, the discussion will be focused on aptamer-based portable colorimetric assays.

#### 3.1.1. Enzyme-Based Colorimetric Aptasensors

**Figure 2:**
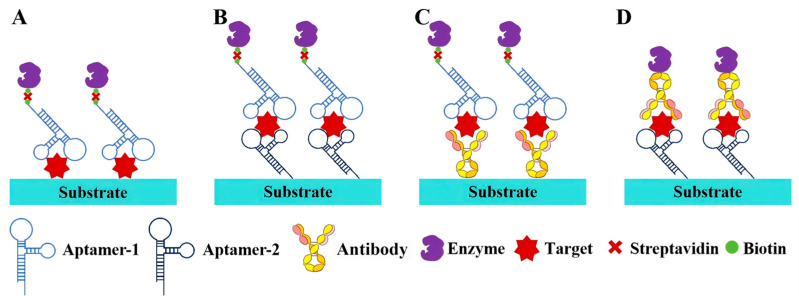
Strategies for aptamer-based enzyme-linked immunosorbent assay (ALISA). (**A**) Direct strategy; (**B**) dual-aptamer strategy; (**C**) aptamer–antibody strategy with aptamer as reporter ligand; (**D**) aptamer–antibody strategy with aptamer as capture ligand.

**Figure 3:**
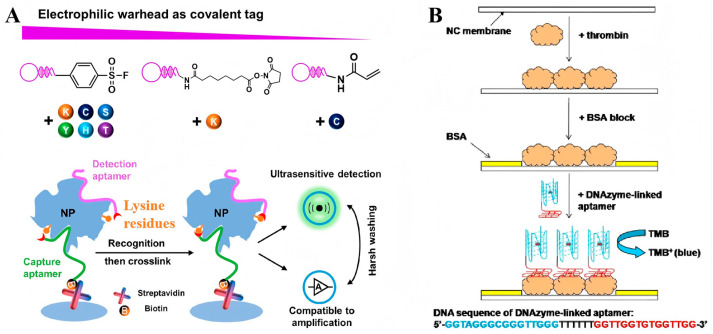
(**A**) Covalent aptamer-based strategies for detection and functional blocking of target protein by specific proximity-mediated cross-link [43]. (**B**) Scheme for the detection of thrombin based on the dot-blot DNAzyme-linked aptamer assay [44]. (Figure was re-printed with permission from the publisher).

Aptamers that are resistant to temperature-induced denaturation possess a significantly longer shelf-life and do not have stringent requirements for delivery and storage, making them an excellent alternative to antibodies. Based on this, a range of colorimetric assays, such as aptamer-linked immunosorbent assays or enzyme-linked aptamer assays, have been developed, utilizing aptamers as the molecular recognition element (Figure 2) [39,40]. The indirect and direct methods are two common designs in enzyme-linked immunoassays. The indirect method, also termed the competitive method, involves immobilizing the target antigen on a solid-phase substrate. In this approach, the target molecules in the sample compete with the immobilized antigen for binding to enzyme-labeled aptamers, resulting in a signal intensity inversely proportional to the antigen concentration in the sample. In contrast, the direct method employs a pair of aptamers that simultaneously bind to distinct epitopes of the target antigen. One aptamer is immobilized on the substrate as a capture ligand, while the other is enzyme-labeled to generate a colorimetric signal. Consequently, the amount of enzyme-labeled aptamer bound to the substrate (i.e., the final signal intensity) is directly proportional to the antigen concentration in the sample. Moreover, an antibody–aptamer pair strategy has also been developed to overcome the limited aptamers that bind two different site on the same target (Figure 2C,D). Surprisingly, numerous studies have confirmed that aptamer-based assays display comparable or even superior performance to antibody-based assays. For instance, Kiel et al. developed an aptamer-linked immobilized sorbent assay (ALISA) for the detection of Francisella tularensis subspecies, and the results demonstrated a higher sensitivity than the antibody-based enzyme-linked immunosorbent assay (ELISA) [41]. In 2014, Wu et al. exploited the competitive interaction between aptamers and antibodies targeting the P48 protein to develop a competitive enzyme-linked aptamer assay (ELAA) for the detection of Mycoplasma bovis (M. bovis). Briefly, the M. bovis-specific antibody binds to the P48 protein immobilized on 96-well plates, thereby inhibiting the subsequent binding of biotin-modified aptamers. Consequently, the aptamers cannot conjugate with streptavidin-horseradish peroxidase (SA-HRP), and so leading to a weakened chromogenic reaction [42]. Recently, a covalent aptamer strategy for sensitive SARS-CoV-2 detection was devised to further augment the binding affinity (Figure 3A) [43]. This strategy involves conjugating aptamers with specific electrophilic warheads to create covalent aptamers. Once the aptamers bind to their target proteins, the electrophilic warheads react with nearby nucleophilic groups such as lysine (K), cysteine (C), serine (S), tyrosine (Y), histidine (H), and threonine (T). The establishment of covalent conjugation significantly strengthens the interaction between aptamers and their targets, leading to superior performance compared to commercial ELISA kits.

Dot-blotting has been a routinely employed point-of-care format with a long-standing history. Similarly to ALISA, aptamers have been extensively employed as recognition components that bind to specific antigens immobilized onto nitrocellulose (NC) or polyvinylidene fluoride (PVDF) membranes. In a typical example, Ying and colleagues developed an aptamer-based dot-blot assay for IgE detection, utilizing a biotinylated anti-IgE aptamer. This method exhibited a linear response relationship within the concentration range of 50 nmol/L to 1 µmol/L. Moreover, a limit of detection as low as 2.89 nmol offered a rapid and cost-effective approach for the diagnosis of topical dermatitis [39]. In addition to enzyme proteins, Wang and his team have developed a novel dot-blot assay for protein analysis, utilizing aptamers and DNAzymes as recognition and signal transduction elements, respectively (Figure 3B). In this method, a peroxidase-mimicking DNAzyme is conjugated with biotinylated thrombin-specific aptamers. When aptamers bind to their targets, the DNAzyme is immobilized onto the membrane, catalyzing a chromogenic reaction to produce a blue dot that can be easily visualized by the naked eye, which offers a sensitive and straightforward means for protein analysis [44]. In another study, streptavidin-modified gold nanoparticles were utilized as a chromogenic agent for dot-blotting analysis of human cardiac troponin I. The binding of aptamer–Au NPs conjugates to immobilized targets resulted in the formation of concentration-dependent red dots. The detection limit of this aptamer–Au NPs-based assay was as low as 5 ng/mL, offering a simple and rapid method for diagnosing myocardial injury [45].

#### 3.1.2. Gold Nanoparticle-Based Colorimetric Aptasensors

The intrinsic localized surface plasmon resonance (LSPR) endows noble metal nanoparticles with excellent optical properties. Particularly, gold nanoparticles (Au NPs) with an absorption spectrum in the UV–visible region of the electromagnetic spectrum, have been extensively utilized for the development of colorimetric assays [46]. In 1997, Mirkin and his colleagues developed a mercaptoalkyloligonucleotide-modified gold nanoparticle probe for highly selective colorimetric detection of polynucleotides [47]. When single-strand target oligonucleotides were incubated with the Au NP probes, a polymeric network of Au NPs formed, accompanied by a color change from red to pinkish/purple. This color change can be readily distinguished by the naked eye, allowing for rapid identification of target polynucleotides without the need for instrumentation. In 2004, Rothberg and his colleagues discovered that single- and double-stranded oligonucleotides have different tendencies to adsorb on gold nanoparticles [48,49]. In brief, the single-stranded but not double-strand oligonucleotides can interact with Au NPs through noncovalent interactions, thereby protecting them from salt-induced aggregation [50]. This mechanism has been extensively utilized for developing rapid colorimetric assays that exhibit femtomole sensitivity and specificity for detecting single-base-pair mismatches [51,52].

**Figure 4:**
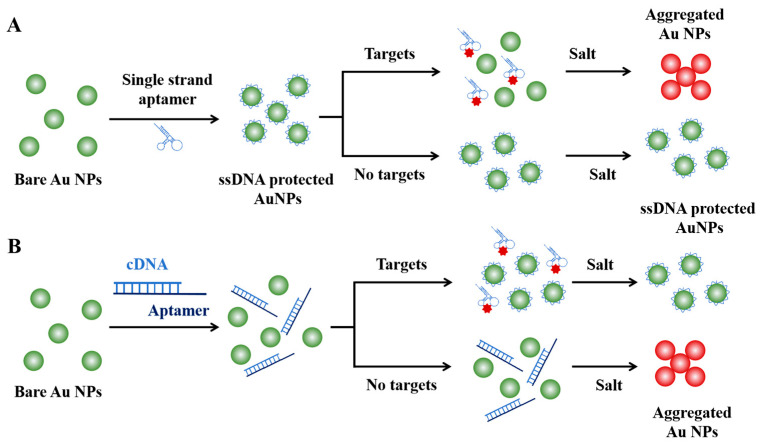
Strategies for aptamer-based colorimetric assays. (**A**) Target-induced Au NPs aggregation strategy. (**B**) Target-inhibited Au NPs aggregation strategy.

Due to the broader target range of aptamers, the integration of aptamers into the Au NPs-based colorimetric assays has further broadened the detectable target from nucleic acids to small molecules or ions, macromolecules, and even entire cells or microorganisms [53,54] (Figure 4). Take dopamine as a paradigmatic example of small molecules: Yang et al. developed a highly sensitive and selective colorimetric aptasensor for dopamine detection [55]. In this assay, the interaction between aptamers and their target molecules triggers a conformational shift from a disordered coil to a stable tertiary structure, which induce the desorption of single-strand DNA aptamers from the surface of Au NPs. This facilitates the salt-induced aggregation of AuNPs, resulting in a discernible color change from red to blue. This aptasensor showed a linear range spanning from 0.54 to 5.4 µM, and the limit of detection (LOD) was 0.36 µM. In 2016, Yi and his colleagues further improved this strategy by developing a dual-mode sensing system through the modification of aptamers with fluorophores [56]. In the absence of dopamine, the absorption of single-strand aptamers on Au NPs protect it from salt-induce aggregation and quench the fluorescence signal. Upon incubation with dopamine-containing samples, the binding-induced desorption of aptamers leads to the aggregation of Au NPs, which subsequently results in a color change from red to blue and fluorescence signal recovery. The fluorescent signal was well suited for samples with intrinsic color that could influence the observation of the color change of Au NPs and displayed higher sensitivity, with a LOD of 78.7 nM.

**Figure 5:**
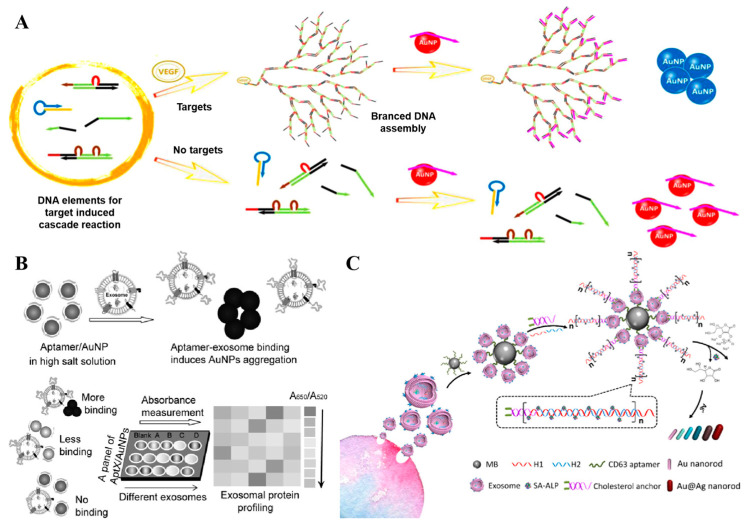
(**A**) Schematic illustration of target protein-induced cascade DNA branched amplification reaction and AuNPs-based colorimetric assay [58]. (**B**) Working principle of the aptamer/AuNP complex for molecular profiling of exosomal proteins [64]. (**C**) Schematic illustration of the mechanism for multicolor visual detection of exosomes based on HCR and enzyme-catalyzed metallization of Au NRs [65]. (Figure was re-printed with permission from the publisher).

In addition to small molecules, the colorimetric aptasensors have also been exploited for the rapid analysis of protein biomarkers. C-reactive protein (CRP), a well-recognized biomarker for inflammatory-related diseases, has found extensive application in clinical diagnosis. Silva et al. have developed an aptamer-based colorimetric assay to facilitate rapid and sensitive CRP analysis. When CRP is present, the guanine-rich aptamer forms a quadruplex structure, leading to the detachment of aptamers from the surface of Au NPs and their preferential binding to the target protein. Subsequently, as they lose the protective effect from the single-stranded aptamers, the unprotected Au NPs undergo a salt-induced aggregation, accompanied by a color change from red wine to blue-purple [57]. Over the past decades, this method has been extensively utilized for detecting protein biomarkers in various types of samples, including blood, urine, saliva, and other samples. Furthermore, Lin and his team have further enhanced the sensitivity of this strategy by coupling the colorimetric assay with a target-assisted cascade amplification reaction (Figure 5A). In their study, an isothermal DNA cascade reaction system comprising a bifunctional hairpin probe, two double-stranded substrate DNA, and two single-stranded auxiliary DNA was designed. The presence of targets initiates the cascade reaction which consumes the single-stranded auxiliary DNA that protect Au NPs, and subsequently induce the salt-induced aggregation and color change. Notably, the detection limit of this assay for VEGF protein is 185 pM, which is lower than that of other aptamer-based detection methodologies [58].

Exosomes, which encapsulate a substantial portion of the phenotypic information of their originating cells, have emerged as a novel class of biomarkers. Accurate identification of exosomes provides new possibilities for disease diagnosis and treatment. Recently, aptasensors have gained prominence as a pivotal tool for detecting exosomes [59,60,61,62,63]. For instance, our group devised a colorimetric profiling strategy for exosomal proteins by utilizing a panel of aptamers specifically designed to target various proteins. The capability to differentiate and profile subtle proteins on exosomes paves the way for more precise diagnosis and molecular subtyping (Figure 5B) [64]. In 2019, Xu et al. developed an aptamer-based dual-signal amplification strategy for the sensitive and multicolor visual detection of exosomes [65]. Firstly, the CD63-specific aptamer-decorated magnetic beads were employed to capture and isolate exosomes. Then, the cholesterol-modified DNA probes were spontaneously inserted into the exosomal lipid membrane, initiating a chain hybridization reaction between the biotinylated H1 and H2 oligonucleotides. Finally, the resultant nano structures were loaded with streptavidin–alkaline phosphatase, which catalyzed the deposition of a silver shell on the gold nanorods (Au NRs), and so resulted in a multicolor change that could be distinguished by the naked eye. Owing to the dual-signal amplification of the hybridization chain reaction and the enzyme catalyzed metallization of Au NRs, highly sensitive detection for exosomes was achieved with detection limits as low as 9 × 10^3^ particles/μL by naked eye and 1.6 × 10^2^ particles/μL with the help of UV-vis spectroscopy (Figure 5C).

Even more excitingly, the colorimetric assay based on Au NPs can also be utilized for the analysis of live microorganisms or cells. In 2008, our group developed the first aptamer-based colorimetric assays specifically designed for cancer-cell detection. Herein, aptamers were covalently conjugated with Au NPs through an Au-S interaction. The binding of aptamers to their targets that overexpressed on cancer cells induced the aggregation of Au NPs on the cell surface, resulting in a distinctive color change from wine red to dark blue, which was accompanied by a red shift in both the absorption and scattering spectrum [53]. Recently, Sun and colleagues successfully isolated a specific aptamer for Escherichia coli using iterative cycles of centrifugation-based partitioning. They further developed an innovative aptamer-based dual-signal amplification colorimetric strategy for bacterial detection. In this study, a sophisticated strand displacement-initiated molecular cascade reaction network was meticulously designed. Upon binding to their targets, the aptamers triggered the release of the elicitation chain (DNA 1), which subsequently interacted with probe 1 to liberate DNA 3. Subsequently, the liberated DNA 3 hybridized with DNA 4 and DNA 5 on probes 2 and 3, respectively, leading to the aggregation of gold nanoparticles (Au NPs). Owing to this cascade signal amplification strategy, the assay exhibited remarkable sensitivity, detecting Escherichia coli at concentrations as low as 10 CFU/mL, while maintaining high specificity [66].

#### 3.1.3. Aptamer-Based Lateral Flow Assays

Lateral flow assays (LFAs), another prominent example of point-of-care testing, have revolutionized the field of in vitro diagnosis according to its rapid turnaround time, ease of operation, and exceptional affordability. Traditionally, antibodies have served as the primary recognition molecules in LFA products. However, the poor stability and the batch-to-batch variations that require specific storage requirements significantly limit the accuracy rate of the conventional antibody-based LFAs. The advent of SELEX technology and the burgeoning application of aptamers in biomedical research have catalyzed the development of aptamer-based LFAs for highly efficient point-of-care diagnosis [67]. In this section, we provide a comprehensive overview of the recent advancements in aptamer-based LFAs according to the working mechanism.

##### Aptamer-Based Sandwich LFAs

**Figure 6:**
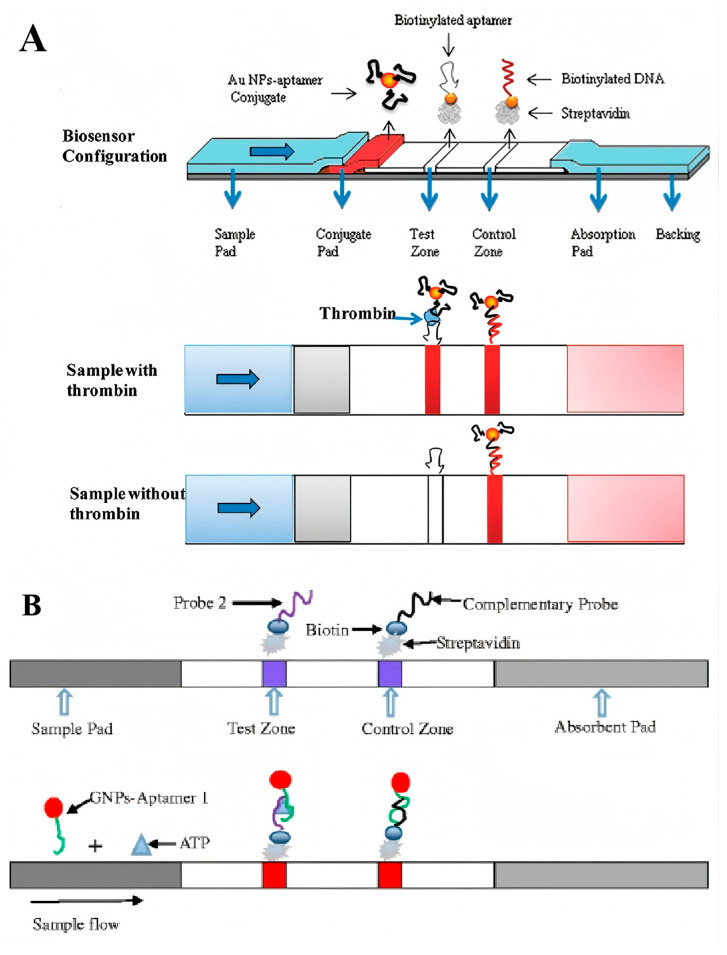
(**A**) Schematic illustration of the configuration and measurement principle of the aptamer-based strip biosensor for thrombin [68]. (**B**) Schematic illustration of sandwich dipstick assay for ATP detection based on split-aptamer fragments [73]. (Figure was re-printed with permission from the publisher).

For analytes with high molecular weights and multiple epitopes, the sandwich structure is the most frequently employed strategy for designing lateral flow assays (LFAs). Typically, the sandwich structure is formed between the target molecules, aptamers conjugated with reporters, and capture aptamers located on the test line. In 2009, Liu and colleagues introduced a dual aptamers-based model system by using thrombin as a detection target (Figure 6A) [68]. In this work, the biotin-modified capture aptamers were immobilized on the test line through streptavidin–biotin binding, and the report aptamers were conjugated with Au NPs through Au-S covalently interaction. After loading the thrombin-containing samples on the sample pad and migrating to the conjugate pad by capillary action, a Au NP–aptamer–thrombin complex was formed and further migrated along the strip to the test zone. The complex was captured by the capture aptamers and resulted in the enrichment of Au NPs on the test line. Then, the excess report aptamer-modified Au NPs migrated to the control line and were captured by the complementary oligonucleotudes, forming another red band. As the most common structure of LFAs, this sandwich strategy with similar designs has been extensively utilized for detecting a diverse array of targets, ranging from small molecules and proteins to nucleic acids, viruses, and even whole diseased cells [69,70].

In addition to dual-aptamer sandwich LFAs, the integration of antibodies and aptamers provides a feasible solution to overcome the limitation posed by the lack of two aptamers which target distinct sites on a target molecule. In 2007, Minagawa and colleagues devised an LFA for α-salivary amylase (sAA) detection by using a 36-mer DNA aptamer (AMYm1–3) as the detection ligand and commercial anti-sAA antibody as the capture ligands. The results of this LFA exhibited a strong correlation with those obtained using an enzyme-linked immunosorbent assay (ELISA) kit, enabling the specific detection of sAA in 0.1% (*v*/*v*) human saliva [71]. While the aptamer–antibody dual ligands strategy broadened the range of detectable targets, identifying an aptamer without competitive interference with the commercial antibody required extensive effort. Furthermore, the incorporation of the unstable and costly antibody undermines the advantages of aptamers. Consequently, the clinical implications of this format warrant further exploration and discussion.

Another reason aptamers are superior lies in the ability for two separate aptamer fragments to recombine their three-dimensional (3D) structure, while maintaining comparable affinity and specificity to the parent aptamer [72]. Based on this property, a split-aptamer-based LFA was designed by conjugating one fragment of the aptamer to the Au NPs and immobilizing the other fragment on the test zone as capture ligands. In 2016, Chen et al. developed a split-aptamer-based sandwich dipstick assay for ATP detection (Figure 6B). In this work, the ATP aptamer was divided into two fragments, aptamer-1 and aptamer-2, which were then modified with a thiol group and biotin, respectively. The report probe was created by conjugating aptamer-1 with Au NPs and loading onto the conjugated pad. Aptamer-2 served as the capture probe that immobilized on the test zone through streptavidin–biotin interaction. Moreover, a complementary ssDNA probe to aptamer-1 was immobilized on the control line. The ATP-containing samples were loaded on the sample pad and migrated along the strip via capillary action. The formation of aptamer-1/ATP/aptamer-2 complexes resulted in a red line appearing on the test zone, while the hybridization between aptamer-1 and the complementary DNA probe generated another red line on the control zone. This split-aptamer-based LFAs demonstrated a linear response across a broad range from 0.5 nM to 5 mM, and exhibited a high degree of specificity for ATP, distinguishing it from other nucleotides such as UTP, CTP, and GTP. Remarkably, the split-aptamer strategy has proven particularly advantageous in detecting small molecules that exhibit weak immunogenicity for antibody development and lack binding sites for dual aptamer binding. Although a comprehensive underlying principle remains elusive, key determinants for refining split-aptamer design have been thoroughly examined, offering pivotal insights of significant reference value [73].

##### Aptamer-Based Competitive LFAs

**Figure 7:**
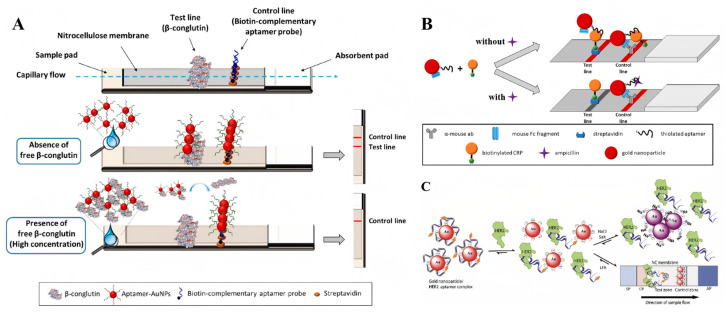
(**A**) Schematic illustration of competitive β-conglutin assay on the strip [74]. (**B**) Schematic illustration of the designed competitive LFA for ampicillin detection [75]. (**C**) Schematic illustration of aptamer-based colorimetric lateral flow assay for human epidermal growth factor receptor 2 [76]. (Figure was re-printed with permission from the publisher).

Competitive assays offer a viable alternative for designing lateral flow assays (LFAs) when dual ligands for a specific target are unavailable. The most prevalent format involves immobilizing target molecules onto the test line, which then compete with free targets present in the sample. When targets are present in the sample, their binding to the aptamers inhibits further interaction between the aptamer and the Au NPs and the immobilized targets on the test line. Consequently, the signal intensity reduces in proportion to the concentration of target molecules in the sample. According to this principle, O’Sullivan and colleagues developed a competitive LFA for β-conglutin analysis (Figure 7A). Herein, the β-conglutin-specific aptamers labeled Au NPs were pre-loaded onto the conjugate pad, while the recombinant β-conglutin was immobilized on the test line. In the absence of target molecules, the aptamer Au NPs migrate to the test line and interact with the immobilized recombinant β-conglutin, producing a distinct red line signal. Conversely, when target molecules are present in the sample, they interact with the aptamers on the conjugate pad, which results in less free aptamers available to interact with the immobilized recombinant β-conglutin, and so a reduced signal will be present in the test line. As reported, this competitive assay could be completed in just 5 min, and a detection limit of 55 pM was achieved [74].

When expanding the competitive strategy for small-molecule detection, the immobilization of small molecules on membranes leads to the inhibition of aptamer–target interactions, as the immobilization process can induce conformational changes in the target molecules. As a result, the modification technology should be meticulously designed and the binding affinity and specificity of aptamers to the immobilized target molecules must be experimentally tested. From another perspective, Lars Kaiser and colleagues have developed a cross-recognition aptamer-based competitive LFA for ampicillin detection (Figure 7B). Firstly, an aptamer that could interact with both ampicillin and C-reactive protein (CRP) was identified by in silico analysis of the sequence homologies between ampicillin and C-reactive protein aptamers. Building on this discovery, a novel LFA was developed by immobilizing the CRP on the test line where it competed with ampicillin molecules in the sample for interaction with aptamers modified on gold Au NPs [75]. Despite providing an alternative strategy for single-aptamer based competitive assay, its performance falls short of clinical application standards, primarily due to the scarcity of cross-recognition aptamers with high binding affinity for both targets. The customized design of SELEX procedures for identifying cross-recognition aptamers is of significant importance to the widespread application of cross-recognition aptamer-based competitive LFA for small-molecule detection.

Additionally, the target-mediated adsorption–desorption of aptamers on Au NPs has also been explored for the design of aptamer-based competitive LFAs. In 2020, Derosa and colleagues introduced a single aptamer-based LFA for HER2 detection that relies on the competitive binding of aptamers between Au NPs and target molecules [76]. In this work, the biotinated aptamers were absorbed on Au NPs through noncovalently interaction. In the absence of HER2 protein, the aptamer–Au NPs complex could be captured by streptavidins that immobilized on the test line and generated a visible color signal. Conversely, when HER2 protein was present in the sample, the binding of aptamers to HER2 protein mediated the release of bare Au NPs that cannot be captured on the test line, and so no color signal could be detected. On the control line, the modification of cationic charged PDDA polymer that interacts with negatively charged Au NPs nonspecifically provides a validation check for the assay system (Figure 7C). Furthermore, a similar strategy was developed by designing an ssDNA probe that partially complemented to the aptamers. The binding of aptamers to their targets released the ssDNA probe, which further mediated the conjugation of Au NPs on the test line [77]. Recently, the potential of this sensing format was further confirmed for dopamine detection in urine samples by another group [78].

### 3.2. Aptamer-Based Fluorescent Biosensing

Fluorescence represents another typical optical signal that has been extensively utilized in biosensing. Its advantages, such as high sensitivity for precise quantification and excellent temporal resolution, have contributed to the widespread application of fluorescent biosensors in clinical settings. In particular, fluorescent aptasensors have found extensive application in the in vitro diagnosis of a diverse array of biomarkers, encompassing nucleic acids, proteins, biotoxins, viruses, bacteria, and even entire diseased cells [79]. Herein, the commonly used fluorescence signal generation strategies will be discussed, including Förster resonance energy transfer-based biosensing, fluorophore-linked aptamer assay, and aptamer-based biosensing with fluorescent light-up probes.

**Figure 8:**
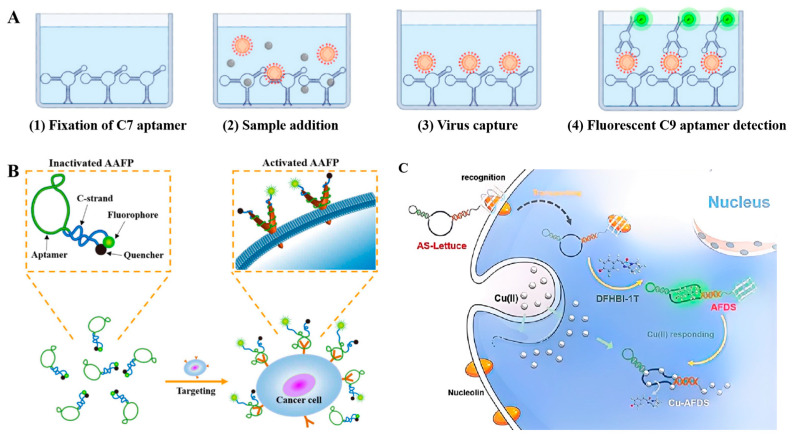
(**A**) Schematic illustration of the FLAA for SARS-CoV-2 S-protein detection [80]. (**B**) Working principle of ‘activatable’ aptamer-based fluorescence probe for the detection of HepG2 cells [81]. (**C**) Schematic illustration of aptamer-functionalized fluorescent DNA sensor for Cu(II) [87]. (Figure was re-printed with permission from the publisher).

The fluorophore-linked aptamer assay (FLAA) is similar to ELISA in that it involves a sandwich structure formed between two ligands and target molecules. In this context, fluorophore-conjugated aptamers serve as the signal output elements, while the capture ligands can be aptamers, antibodies, or other high affinity ligands. In 2022, Alvarez-Salas introduced a FLAA for SARS-CoV-2 spike-protein detection by using the C7/C9 aptamer pair [80]. Firstly, the spike proteins were captured by C7 aptamers covalently immobilized on the surface of 96-well plates. After washing with TNa7 buffer, the FAM-labeled C9 aptamers were added and incubated. Subsequently, excess aptamers were removed through buffer replacement. Finally, the fluorescence signal, which positively correlates with the target concentration, was recorded (Figure 8A). Due to its high similarity to ELISA, this method has been adapted to detect a wide range of target molecules.

Förster resonance energy transfer (FRET) is a process that occurs between adjacent donor and acceptor fluorophores when their emission and absorption spectra overlap. As the efficiency of FRET is significantly influenced by the distance between the two fluorophores, the distinct structure switch of aptamers, mediated by target binding, has been widely applied for aptamer-based fluorescent biosensor design. In 2007, Lai et al. reported an activatable aptasensor for the detection of HepG 2 cells [81]. Initially, the TLS11a aptamer specific to HepG 2 cells was identified through Cell-SELEX. Subsequently, two short-extending complementary DNA sequences on the 3′ and 5′ terminus of aptamers was designed to form a hairpin structure. As shown in (Figure 8B), in the absence of a target, the forming of a hairpin structure brings the fluorophore and quencher together, quenching the fluorescence signals. However, upon introduction of HepG2 cells, binding of the aptamers to their targets on the cell surface disrupts the hairpin structure and so releases the fluorescence signal. Soon after, this strategy was further improved by measuring the fluorescence ratio between the donor and acceptor fluorophores. In 2020, Sapkota and colleagues reported an aptasensor for lysozyme detection using FRET between the donor Cy3 and the acceptor Cy5 [82]. Based on a similar design, Aissa et al. achieved highly sensitive quinolone antibiotic ofloxacin detection by modifying aptamers with FAM and TAMRA at the 3′ and 5′ terminus, respectively [83]. These advancements demonstrate the versatility and potential of FRET-based aptasensors for detecting a wide range of targets.

Light-up aptamers are aptamers that can stimulate the generation of fluorescence signals from non-fluorescent molecules through binding interaction. In 1999, Wilson and Grate discovered an RNA aptamer with high affinity and specificity to malachite green (MG) molecules, and it significantly enhancied the fluorescence properties of MG molecules [84]. In 2011, Jaffrey and colleagues reported another RNA aptamer that induced fluorescence in HBI derivative or thiazole orange derivative with higher biocompatibility [85]. Subsequently, a type of light-up aptamer, known as Spinach, was identified and applied in biosensing and bioimaging. The primary advantage of light-up aptamer sensors is the low background signals. In 2021, Sett et al. reported an aptasensor for theophylline detection by combing a binding aptamer with a light-up aptamer [86]. In this work, the binding of theophylline to the aptamers mediated a structure switch through loop-to-loop interaction, enabling the binding of MG to the light-up aptamers, and subsequently generating a fluorescent signal. More recently, Mou et al. developed a light-up aptamer-based biosensor for Cu^2+^ ion analysis [87]. The abundance of Cu^2+^ ions interacts with the nucleobase and affects the secondary structure of the aptamer, resulting in the release of DFHBI-1T molecules and the reduction of fluorescence signal (Figure 8C).

In addition to organic fluorophores, fluorescent nanomaterials such as quantum dots, upconversion nanomaterials, and long-afterglow materials have been employed in fluorescence biosensing due to their high quantum yield, low background, and excellent photostability. Since these nanomaterials operate on similar sensing mechanisms to organic fluorophores, they will not be discussed in detail in this section. However, it is worth noting that many researchers still rely on fluorescence spectrophotometers or microplate readers to monitor fluorescence signals. The development of portable fluorescence-detection instruments has lagged behind the progress of detection kits. Therefore, greater attention should be directed towards the development of fluorescent detection devices that are user-friendly and cost-effective, thereby facilitating the application of fluorescence-detection technology in primary healthcare and even home healthcare settings.

## 4. Aptamer-Based Electrochemical Biosensors

Electrochemical biosensors are devices that convert the biological interactions occurring on the electrode surface into readable electrical signals. These sensors have garnered significant attention and found widespread application in the field of point-of-care testing (POCT) due to their remarkable advantages, including high sensitivity, strong specificity, rapid response, and the capability to provide real-time quantitative information [88,89]. Various portable electrochemical biosensors for blood glucose, uric acid, and ketones analysis have been commercialized and have become widely used in at-home health monitoring. In particular, the properties of aptamers, including high sensitivity, portability, miniaturization capabilities, and quantitative detection, are taken into consideration for the development of electrochemical systems [90,91]. This section will delve into the combined application of nucleic acid aptamers with traditional electrochemical detection methods, such as amperometry, voltammetry, and potentiometry. Furthermore, the latest research advancements in novel electrochemical detection methods, including field-effect transistors and wearable electrochemical devices, will also be presented. These advancements are poised to revolutionize the field of electrochemical biosensing, making it even more accessible and effective for a wide range of applications.

### 4.1. Traditional Electrochemical Sensing

Amperometric biosensors operate by measuring the current signal generated by the redox reaction occurring on the electrode surface under a constant voltage between the working electrode and the counter electrode. In 2020, Jiang et al. developed a DNA nanotetrahedron-assisted aptasensor for exosomal protein profiling. In this study, aptamer-decorated DNA nanotetrahedrons were covalently modified onto a gold electrode, enabling aptamers to bind to targets with an optimal orientation through the modulation of microscopic interface modification on the electrode surface. After the target exosomes were captured by immobilized aptamers, another Au NP-based nanoprobe modified with polyA-aptamers and polyA-DNA-biotin was incubated as the signal amplification element. The large amount of biotin on the surface of the nanoprobe can significantly increase the ratio of horseradish peroxidase (HRP) to the target, thereby enhancing the output of electrochemical signals. The results indicate a detection limit of 1.66 × 10^4^ particles/mL for HepG2 liver cancer exosomes [92]. Considering the high cost of gold electrodes, carbon-printed electrodes present a better option for electrochemical biosensing. For instance, Petroni et al. realized sensitive and specific detection of nitrite and ascorbate by using screen-printed electrodes [93]. Notably, the modification of electrode materials plays a crucial role in the intensity of electrochemical signals. For example, screen-printed carbon electrodes coated with a carboxyethylsilanetriol-modified graphene oxide derivative were employed for the detection of cardiac troponin I (cTnI), achieving a detection limit of 0.6 pg/mL. In another study, Yunus and colleagues fabricated a diazonium-grafted screen-printed carbon electrode for the detection of CFP10 and MPT64 antigens. The detection limits for CFP10 and MPT64 were as low as 1.68 ng/mL, respectively, providing an efficient approach for early tuberculosis diagnosis [94].

Voltammetry, or cyclic voltammetry, involves applying a pulsed voltage to a closed circuit formed by a working electrode and counter electrode. By varying the potential at the interface between the working electrode and the electrolyte at a specific rate, active substances on the working electrode undergo oxidation/reduction reactions. The magnitude of the response current generated by the electrochemical reactions occurring at the electrode is proportional to the concentration of electro-active substrates. Moreover, the integration of current and potential difference enables a responsive system to be applied as a multicomponent detector. For instance, Sanghavi developed a microfluidic-based electrochemical sensor for cortisol detection, an important hormone with various physiological functions. In this work, the electro-active triamcinolone, which has a similar molecular structure to cortisol, was used to construct the competitive assay. Firstly, the aptamers modified on Au NPs were pre-bound with triamcinolone. After being incubated with cortisol, the triamcinolones were released and detected by using square wave voltammetry. The results showed a broad linearity range from 10 μg/mL to 30 pg/mL and exhibited high specificity for cortisol compared to other glucocorticoids, such as estradiol, testosterone, and progesterone [95]. In 2019, Adeel and colleagues developed an electrochemical assay for myoglobin analysis by designing novel 2D materials with large bandgaps. Herein, the boron nitride nanosheets were exfoliated and spin-coated onto the fluorine-doped tin oxide (FTO) electrode, followed by the deposition of Au NPs. The Au NPs provide both modification sites for aptamers and excellent catalytic sites for electrochemical reactions. This multilayered electrode displayed sensitive responses for myoglobin molecules, with a detection limit of 34.6 ng/mL, indicating high potential for POC diagnosis in clinical samples [96]. Recently, paper-based electrochemical biosensors have received significant attention for POC diagnosis due to their additional advantages, including low cost and disposability [90,97].

The principle of a potentiometric sensor primarily relies on the specific interaction between the sensor and the analyte. When a local Nernstian equilibrium forms at the sensor interface, there is no current flow through the system, and so the electrochemical response is proportional to the concentration of analytes. Compared to amperometric biosensors, potentiometric biosensors do not consume the target species, demonstrating significant application prospects in non-destructive analysis. Over the past decade, this strategy has been extensively utilized for the detection of cancer cells and bacterial cells [98]. For instance, Zelada-Guillén developed an aptasensor for rapid and ultrasensitive bacteria detection by using carbon nanotubes as the transducer layer and exploiting aptamers as biorecognition elements. These aptasensors successfully achieved high-performance analysis of targets including *S. typhi*, *E. coli*, and *S. aureus* by using corresponding aptamers [99]. However, the immobilization of aptamers on carbon nanomaterials is a time-consuming and labor-intensive process that significantly impacts the performance of aptasensors. To address this issue, Ding et al. reported a label-free potentiometric strategy for rapid, sensitive, and specific detection of Listeria monocytogenes using a polycation-sensitive membrane electrode. In the absence of the target bacterium, the negatively charged aptamers interact electrostatically with protamine, inhibiting the electrochemical signals. When incubated with target-containing samples, the binding of aptamers to the targets prevents this electrostatic interaction with protamine, thereby restoring the electrochemical signals. The high performance of this assay, with a detection limit as low as 10 CFU/mL, demonstrates its high potential for determining and identifying trace levels of pathogens in real environmental samples [100].

### 4.2. Innovative Electrochemical Sensing

Over the past decade, advancements in electrochemical biosensing have been driven by the development of innovative electroactive materials and their integration with other cutting-edge technologies [101]. Notable examples encompass transistor biosensors exhibiting ultra-high sensitivity, automated electrochemical systems integrated with microfluidics, and wearable electrochemical biosensors designed for real-time monitoring [102,103,104,105]. While recent comprehensive review articles have systematically covered each of these domains, this section will focus on introducing some representative and illustrative cases, aiming to impart a general understanding of these emerging development trends.

**Figure 9:**
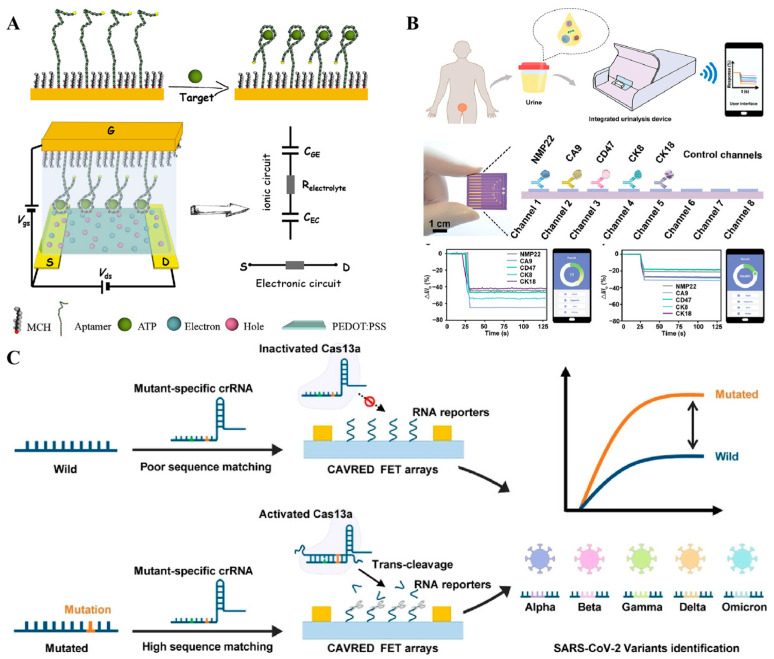
(**A**) Schematic illustration of the aptamer-based interdigitated organic electrochemical biosensors [108]. (**B**) Schematic of integrated urinalysis device for target detection from urine samples [110]. (**C**) The working principle of CRISPR-based amplification-free viral RNA electrical detection platform for SARS-CoV-2 variants identification [111]. (Figure was re-printed with permission from the publisher).

Field-effect transistor biosensors, also referred to as semiconductive biosensors, employ thin-film transistors comprising gate, drain, and source electrodes, with a semiconductor film positioned between the drain and source electrodes. The sensing mechanism hinges on a sensitive alteration in the potential drop or capacitance at the gate–electrolyte or channel–electrolyte interface, which in turn modulates the channel current. Due to their ultra-high sensitivity, field-effect transistor biosensors have exhibited substantial application potential in the realm of biosensing [106,107]. In 2019, Liang and his team developed an aptasensor based on an organic electrochemical transistor for the detection of adenosine triphosphate (ATP) (Figure 9A) [108]. Compared to traditional amperometric sensors, this aptasensor boasts a detection limit that is four orders of magnitude lower, reaching approximately 10 picomolar (pM). For oligonucleotide biomarkers, Lin and her colleagues created an organic electrochemical transistor integrated with flexible microfluidic systems. In this setup, the hybridization of target DNA with complementary probes immobilized on the semiconductor film triggers a change in channel current that is proportional to the concentration of the analyte. In addition to organic semiconductors, inorganic transistors have also been employed in the construction of biosensors. Our research group has developed an indium gallium zinc oxide (IGZO) field-effect transistor biosensor for the analysis of proteins and nucleic acids [109]. In this study, we implemented a controllable modification strategy to regulate the orientation of antibodies within the channel, thereby enhancing their binding efficiency with target molecules. Furthermore, we constructed an integrated device for point-of-care diagnosis. By integrating this device with a machine-learning algorithm, we achieved an accuracy of 95.0% in identifying bladder cancer using five protein biomarkers [110] (Figure 9B). When coupled with CRISPR-based amplification, the device could also be utilized for profiling viral RNA at single-nucleotide resolution (Figure 8C) [111]. Through the design of a sensing array, the transistor could also be seamlessly integrated into miniaturized devices without compromising performance, enabling multi-target, high-throughput analysis.

**Figure 10:**
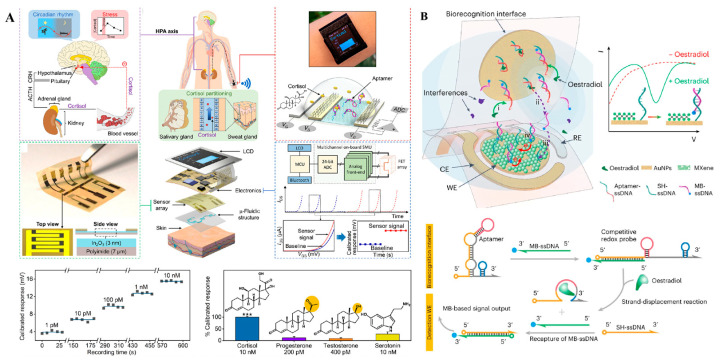
(**A**) Working principle of wearable aptamer-field-effect transistor sensing system for noninvasive cortisol biomarker monitoring [115] (***: *p* < 0.001). (**B**) Schematic illustration of wearable nanobiosensor based on strand-displacement aptamer switch for non-invasive reagentless female reproductive hormone analysis [116]. (Figure was re-printed with permission from the publisher).

Real-time monitoring of physiological states holds immense significance in understanding disease dynamics, and the advent of wearable biosensors has brought this goal closer to reality [112,113]. Recently, wearable blood glucose meters have been commercialized for continuous glucose monitoring, showcasing the potential of electrochemical sensors in the development of wearable biosensors. Additionally, aptamers offer several advantages over antibodies, including high affinity and stability, a broader target range, and greater economic efficiency. These qualities render aptamers both effective and economical choices for implantable and wearable applications [114]. In 2022, Wang and his colleagues developed an aptamer-based wearable field-effect transistor sensor for noninvasive cortisol monitoring [115] (Figure 10A). In this study, a nanometer-thin film of In_2_O_3_ was deposited on polyimide-based flexible substrates, and cortisol-specific aptamers were immobilized onto it through covalent interactions. When the aptamers bind to their target cortisol, it induces conformational rearrangements in the negatively charged aptamer phosphodiester backbones, generating measurable electronic signals through surface charge perturbations. Thanks to its ultrahigh sensitivity, this autonomous and wireless sensor enables noninvasive and real-time cortisol monitoring in sweat, providing a more comprehensive view of an individual’s physiological status. Recently, Ye et al. reported a wearable aptasensor for non-invasive in situ monitoring of oestradiol (Figure 10B) [116]. By utilizing a gold nanoparticle-MXene-based working electrode, they achieved extraordinary sensitivity, with an ultra-low limit of detection of 0.14 pM, without the need for any additional signal amplification. In this study, sweat was generated through iontophoresis and collected using integrated capillary bursting valves. The binding of oestradiol to the aptamers triggered a strand-displacement reaction, ultimately leading to the enrichment of methylene blue-tagged ssDNA on the working electrode. This enrichment generated measurable electrochemical signals that were proportional to the concentration of oestradiol. The results demonstrated a high correlation between sweat and blood oestradiol levels, showcasing the potential for non-invasive hormone monitoring through sweat samples.

Overall, aptamer-based biosensors have significantly enhanced electrochemical biosensing capabilities, particularly for the analysis of specific small molecules. In addition to serving as biorecognition elements, aptamers can also function as signal transduction and amplification elements through strand displacement reactions or target-induced conformational switching. The integration of aptamers with novel electrochemical sensing systems further broadens their application in the field of point-of-care (POC) diagnosis, allowing their advantages in various aspects to be more fully demonstrated. Correspondingly, the application of aptamers expands the range of detectable targets for wearable electrochemical devices and even further enhances their detection sensitivity.

## 5. Conclusions and Future Prospective

Over the past 30 years of rapid development, significant breakthroughs have been made in the screening, synthesis, and modification processes of aptamers, paving the way for their widespread application. Aptamers exhibit numerous advantages, including stability, uniformity, and cost-effectiveness, which make them highly promising for biosensing applications, particularly in the field of point-of-care testing (POCT). Many aptamer-based biosensors utilizing various methodologies have already been approved for clinical use or are currently in clinical trials.

However, the number of aptamer-based products still lags behind antibody-based tests. This can be attributed to several reasons [117]. Firstly, the controllability of aptamer screening needs further improvement, as quickly obtaining aptamers targeting different analytes or epitopes on the same analyte is crucial for widespread application. Secondly, the absence of unified standards for aptamer screening and characterization hinders the widespread adoption of aptamers developed by different research groups. Moreover, discrepancies between screening environments and practical application environments can significantly diminish their clinical performance, and, currently, only extensive and labor-intensive validation experiments can assess their performance on clinical samples. Furthermore, the stability of aptamer secondary structures is pivotal for their affinity. Finding methods to maintain aptamer conformations over extended periods and reduce their dissociation constants is vital for enhancing detection sensitivity and result reproducibility. In the context of portable POCT, the development of aptamer-based test kits has surpassed the progress in portable device development. The current challenge lies in the high manufacturing cost. Therefore, reducing device costs and increasing detection throughput is essential for achieving widespread application.

Despite the array of challenges currently impeding the widespread clinical application of aptamers, the ongoing dedication and investment of numerous researchers and R&D enterprises are steadily addressing these engineering hurdles one by one. Notably, during the COVID-19 pandemic, molecular point-of-care testing (POCT) technology has undergone rapid development, and the notion of rapid testing has gained widespread acceptance. Both of these factors have created a conducive environment for the advancement of aptamer-based POCT technologies. It is anticipated that in the coming years, we will witness an increasing number of aptamer-based products being utilized in clinical settings.
